# Distribution and genetic diversity of adeno-associated viruses in bats from coastal areas of Southeast China

**DOI:** 10.1038/s41598-020-60721-z

**Published:** 2020-02-28

**Authors:** Changqiang Zhu, Chunhui Wang, Jiahong Wu, Fuqiang Ye, Ruichen Lv, Dan Hu, Lele Ai, Lu Yang, Ting Wu, Bo Li, Chenxi Ding, Bin Zhang, Heng Lv, Changjun Wang, Weilong Tan

**Affiliations:** 1 Eastern Theater Command Centers for Disease Control and Prevention, 293 Zhongshan East Rd, Nanjing, 210002 P. R. China; 2 Institute for Disease Prevention and Control of PLA, Beijing, 100071 China; 3 Key Laboratory of Environmental Pollution Monitoring and Disease Control, Ministry of Education, Guizhou Medical University, Guiyang, 550025 China; 4 Jinling Hospital Nanjing, Nanjing, 210002 China

**Keywords:** DNA sequencing, Genome evolution, DNA sequencing

## Abstract

Bats are associated with several important zoonotic viruses from different families. One example includes adeno-associated viruses (AAVs), that are extensively detected in several animals, especially primates. To understand AAVs distribution and genetic diversity in the coastal areas of Southeast China, a total of 415 intestine samples were mostly collected from two provinces of southeast China, i.e., Zhejiang and Fujian province. Intestine samples from five bat species were collected for AAVs detection. The average prevalence rate for AAV detection among these samples was 18.6% (77 positives out of 415 samples) and ranged from 11.8 to 28.9% between the five bat species. This suggests that AAVs are widely distributed in diverse bat populations in southeast coastal areas of China. Based on the genome sequence of bat adeno-associated virus-CXC1(BtAAV-CXC1) from one AAV-positive sample, the genetic diversity of the detected AAVs were assessed and analyzed. Phylogenetic analysis revealed that BtAAV-CXC1 was comparatively distant to other major AAVs from mammals and non-mammals, with only a 52.9~64.7% nucleotide identity. However, they were phylogenetically closer to *Rhinolophus sinicus* bat adeno-associated virus (Rs-BtAAV1), with a 74.5% nt similarity. Partial analysis of the rep and cap overlapping open reading frame (ORF) sequences from bat AAV samples revealed 48 partial rep sequences and 23 partial cap sequences from positive samples shared 86.9 to 100% and 72.3 to 98.8% nucleotide identities among themselves, respectively. This suggests that the detected AAVs had a distinctly high genetic diversity. These findings led us to conclude that diverse AAVs may be widely distributed in bat populations from the southeast regions of China.

## Introduction

Adeno-associated viruses (AAVs) were discovered in 1965 as a small contaminant. AAVs are a group of small single-stranded DNA (ssDNA) viruses without envelopes that belong to the parvovirus family. The entire genome of AAV is approximately 4.7 kb and contains two major ORFs that encode for Rep and Cap proteins. Under normal circumstances, the AAV virus can coexist within the mammalian host for an extended period without apparent pathogenicity. However, productive infection only occurs in the presence of a helper virus, either adenovirus or herpesvirus^1–7^. Due to this, AAV has become an important gene therapy vector for its non-pathogenic and conditional infectious life cycle^8^.

The genetic diversity of AAV is very high among its natural hosts. To date, thirteen AAV serotypes (designated AAV1-AAV13) and more than 100 unique variants have been isolated and identified from primate and non-primate hosts^9–25^. Hosts can be as diverse as Chiropteras, Porcine, Serpentes, Bovines, Caprines and Chickens. Among these natural hosts, bats are more geographically dispersed. In addition, Bats host a greater number of emerging viruses per species compared to any other mammalian clade^26,27^. Bats have been increasingly recognized as a natural host for AAVs in recent years, in addition to the many other important zoonotic viruses, such as SARS, MERS, and Ebola virus^28–31^. With the development of molecular and sequencing technologies, numerous novel AAVs are being discovered. These include BtAAV-YNM^14^, Rp-BtAAV1 and Rs-BtAAV1^32^. Yan Li identified several adenoviruses and AAVs in a wide host range of bat species with remarkable genetic diversity^14^. Lau SKP investigated the presence of parvoviruses among different bat species in Hong Kong and mainland China^32^. Their work broadened the current knowledge of AAV diversity in Bats. Several studies also have investigated the origins of viral diversity in bats or the maintenance of bat-associated viruses in nature. In addition, considerable emphasis has been placed on understanding the direct epidemiological interface from bats to humans^33–35^.

In our previous study, several novel bat-associated viruses in southeast China were annotated through metagenomic sequencing technology^36^, including coronavirus, norovirus, adenovirus, bocavirus, astrovirus, circovirus, and adeno-associated viruses. To date, there have been no adeno-associated virus studies in the coastal areas of Southeast China. The main aim of this study was to understand AAV distribution and genetic diversity in these areas. Our study could enrich the understanding of bats as a pathogen library and provided new insights into the transmission of AAV from bats to humans.

## Results

### Identification and prevalence of AAVs in bats in the coastal regions of Southeast China

**Figure 1:**
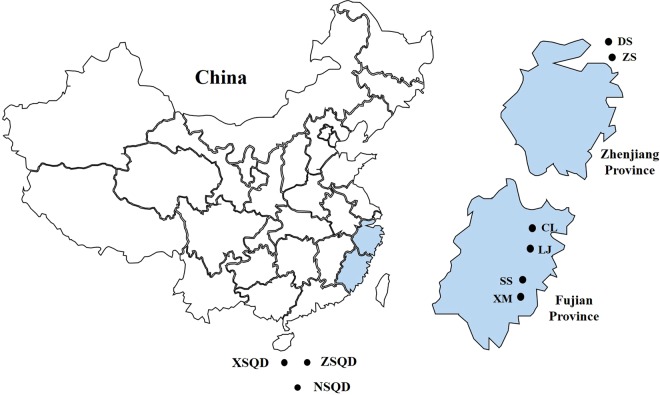
Sampling locations of bats in the two southeast coastal provinces of China. (D.S.: Daishan, Z.S.: Zhoushan, C.L.: Changle, L.J.: Lianjiang, S.S.: Shishi, X.M.: Xiamen, X.S.Q.D.: Xishaqundao, Z.S.Q.D.: Zhongshaqundao, N.S.Q.D.: nanshaqundao).

A total of 415 intestinal tissue samples were collected from five bat species in Zhejiang and Fujian provinces from Southeast China from 2015 to 2018 (Fig. 1). These samples were then used to determine and understand the presence of AAVs. A 1100 bp fragment encompassing the rep and cap regions of known AAV genome was successfully amplified from 77 out of 415 bat samples (18.6%). The prevalence rate ranged from 11.8 to 28.9% and was detected from four insectivorous bat species. AAVs in the study were detected at nearly all sampling locations except in the SS and LJ regions. Among all four bat species, the virus detection rate for *Rhinolophus pusillus* was the highest (4 locations out of 6), followed by *Myotis davidii*, *Rhinolophus ferrumequinum* and *Scotophilus kuhli*. These results demonstrated that AAVs were widely distributed in diverse bat populations in southeast coastal regions of China (Table 1).

**Table 1:** Prevalence of AAVs among the five bat species from the two provinces in China.

Bat species	No. positive/no. tested (%)						
	ZS	DS	XM	CL	SS	LJ	All locations
*Rhinolophus pusillus*	30/102(29.4)	20/91(22.0)	2/19(10.5)	8/44(18.2)	0/35	0/24	60/315(11.8)
*Rhinolophus ferrumequinum*	—	5/29(17.2)	—	—	—	—	5/29(17.2)
*Scotophilus kuhli*	—	—	1/8(12.5)	—	—	—	1/8(12.5)
*Myotis davidii*	7/27(25.9)	—	—	4/11(36.4)	—	—	11/38(28.9)
*Myotis formosus*	—	0/25	—	—	—	—	0/25
All species	37/129(28.7)	25/145(17.2)	3/27(11.1)	12/55(21.8)	0/35	0/24	77/415(18.6)

^*^ZS: Dinghai; DS: Daishan; XM: Xiamen; CL: Changle; SS: Shishi; LJ: Lianjiang.

### Nearly complete genome analysis of BtAAV-CXC1

**Figure 2:**
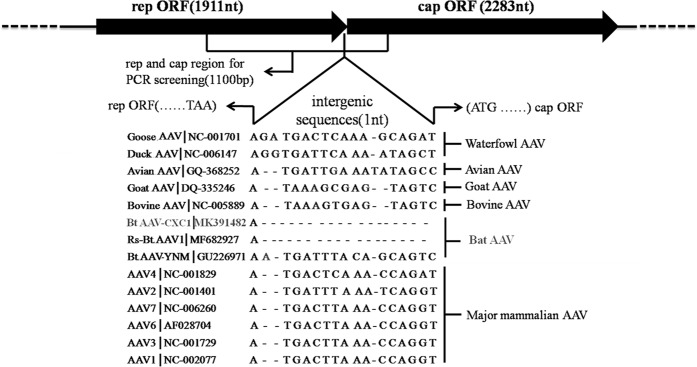
The genome of BtAAV-CXC1. Schematic diagram demonstrating the relative scale of rep and cap ORFs, and the intergenic sequence of BtAAV-CXC1. The region selected for PCR is indicated. Intergenic sequence alignment of BtAAV- CXC1 to those of other AAVs. AAV species are shown left of the alignment and the rough classification is shown on the right of the alignment.

We sequenced one bat AAV strain that was isolated from *Rhinolophus pusillu* sample in Fujian province and designated it BtAAV-CXC1 (GenBank accession number: MK391482). The nearly complete genome of BtAAV-CXC1 was 4263 nt in length with a G + C content of 54.51%, with a 4194 nt coding region and a 1 nt intergenic region. The putative ORFs of rep and cap were predicted to be 1911 and 2283 nt in length, respectively. The Rep and Cap proteins of BtAAV-CXC1 shared an average of 61.0% (ranging from 46.4 to 85.7%) and 27.7% (ranging from 10.7 to 35.5%) pairwise amino acid identity, respectively to the reference AAV genome (Fig. 2). This demonstrated that the genome of BtAAV-CXC1 was obviously different from the reference AAV genome in this study.

### Phylogenetic origin of BtAAV-CXC1

**Figure 3:**
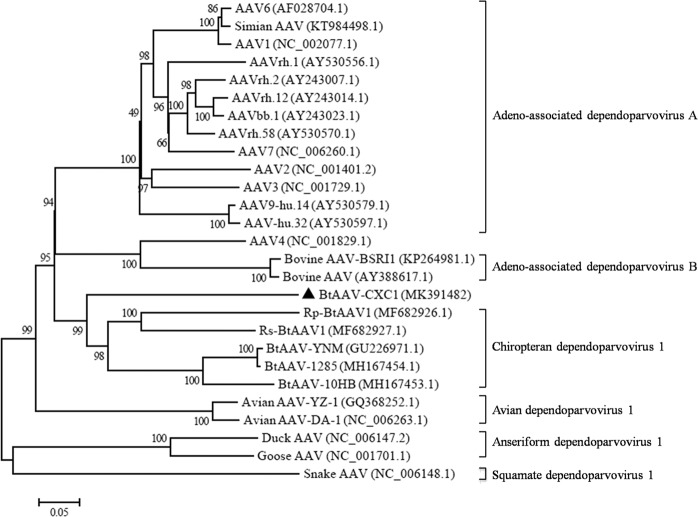
Phylogenetic origin of BtAAV-CXC1. The phylogenic tree was constructed using the maximum likelihood method and the best evolutionary model (GTR + G + I). Bootstrap values were calculated from 1000 trees. The triangles in the phylogenetic trees denote the sequences derived in this study.

In order to investigate the phylogenetic origin of BtAAV-CXC1, a large-scale maximum likelihood (ML) phylogenic tree was constructed with 26 sequences from members of the *Dependovirus* genus (both full length and partial genomes). The ML tree demonstrated that BtAAV-CXC1 was most closely related to Rs-BtAAV1, a previously reported BtAAV from a *Rhinolophus sinicus* bat^32^, with a 25.5% nucleotide difference (Fig. 3). BtAAV-CXC1 was also related to other bat AAVs with a 58.4–77.5% nt identity and was relatively divergent from the majority of AAVs from primates and other animals (i.e., bovines, chicken, waterfowl and snakes), with a 52.9–64.7% nucleotide similarity. These findings indicated that BtAAV-CXC1 was a novel BtAAV species.

### Partial rep and cap sequence analysis of bat AAVs

**Figure 4:**
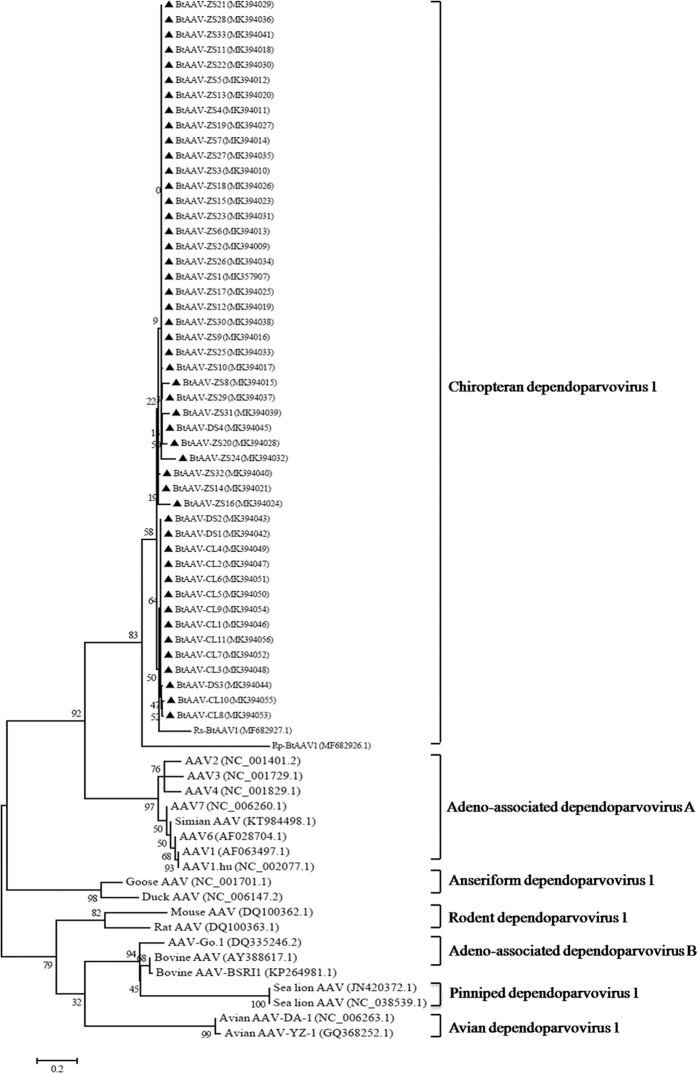
The phylogenetic origin derived from partial rep sequence analysis of bat AAVs. The phylogenic tree was constructed using the maximum likelihood method and the best evolutionary model (GTR + G + I). Bootstrap values were calculated from 1000 trees. The triangles in the phylogenetic trees denote sequences derived in the study.

**Figure 5:**
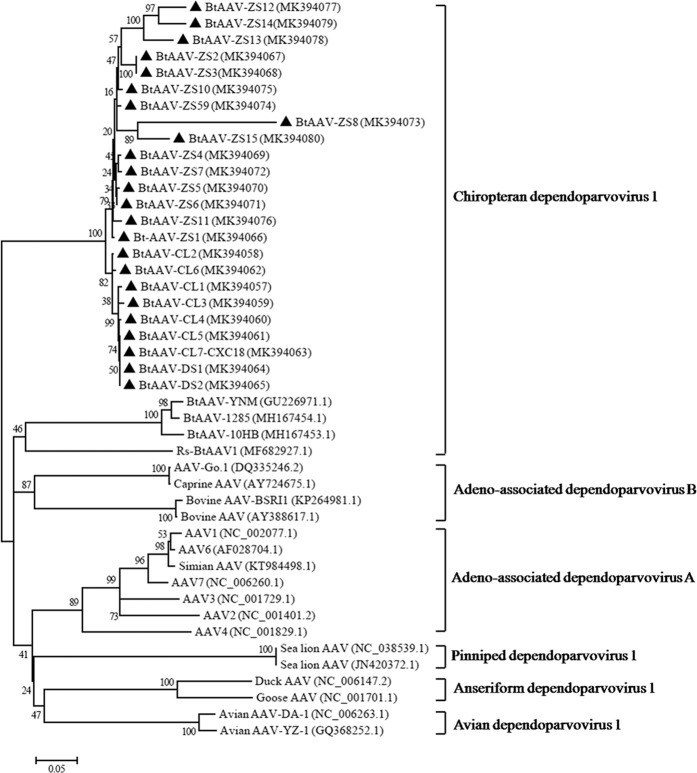
The phylogenetic origin derived from partial cap sequence analysis of bat AAVs. The tree was constructed using the maximum likelihood method and the best evolutionary model (GTR + G + I). Bootstrap values were calculated from 1000 trees. The triangles in the phylogenetic trees denote sequences derived in the study.

To investigate the genetic diversity of bat AAVs, partial rep and cap ORF sequences were obtained through direct sequencing of the PCR products (n = 48 and n = 23; selected from positive samples during the PCR screening assay). The amplified regions covered 1100 nt of the cap region and 841nt of the rep region based on Rs-BtAAV1 (GenBank accession number: MF682927). As shown in Fig. 4, 48 amplicons of the rep region were grouped into bat clade with 86.9 to 100% nucleotide identities between them. In addition, they shared 71.5 to 84.2% identities with other AAVs (Table 2). Further analysis of the 23 partial cap sequences demonstrated that 23 bat AVVs shared 72.3 to 98.8% nucleotide identities among themselves and 59.4 to 72.9% identities with other AAVs (Fig. 5). These results demonstrated that diverse AAVs were widely distributed in bat populations from the southeast coastal regions of China.

**Table 2:** Comparison of AAV genetic diversity in different hosts (pairwise nucleotide similarity based on the cap region).

Location/species	Minimum value**/**Maximum value (Mean value)										
	CL(batAAVs)	DS(batAAVs)	ZS(batAAVs)	Bat	Human	Caprine	Bovine	Sea_lion	Avian	Duck	Goose
CL(batAAVs)	96.9/100.0(99.3)	95.2/100.0(98.2)	87.0/98.8(95.9)	78.3/83.0(81.9)	73.2/8.16(81.7)	74.8/78.0(76.4)	71.9/78.3(75.7)	74.7/82.0(80.3)	78.4/80.8(79.8)	76.5/77.9(77.4)	74.8/76.3(75.7)
DS(batAAVs)	95.2/100.0(98.2)	94.3/100.0(97.0)	87.6/99.9(96.2)	78.6/83.0(81.7)	76.2/84.1(81.7)	75.5/78.6(76.8)	74.1/77.3(75.7)	77.3/81.2(80.1)	75.3/80.8(78.6)	77.9/78.6(78.2)	76.3
ZS(batAAVs)	87.0/98.8(96.2)	87.6/99.9(96.2)	86.9/100.0(98.2)	75.9/82.5(81.8)	73.4/84.1(81.2)	73.3/78.8(77.9)	71.6/77.9(76.6)	75.2/81.9(80.5)	71.5/78.6(77.7)	73.4/78.6(77.9)	76.3

*ZS: Dinghai; DS: Daishan; CL: Changle.

## Discussion

Adeno-associated viruses were detected from the majority of bats collected from the southeastern coastal regions of China. However, no adeno-associated virus was successfully isolated from Vero E6 cells and no genomic sequences of adeno-associated virus were detected in challenge experiments using suckling mice. This may be due to the viral replication of adeno-associated viruses that rely on adenovirus proliferation and expansion.

Four out of the five insectivorous bat species, including *Rhinolophus pusillus*, *Rhinolophus ferrumequinum*, *Scotophilus kuhli*, and *Myotis davidii*, have been reported to be native carriers of diverse AAVs around the southeastern coastal regions of China. The average prevalence rate is as high as 18.6%, which suggests that AAVs are considerably prevalent among various bat species. However, the absence of *Myotis formosus* AAV in this study may be due to the limited sampling size. Partial rep and cap ORF sequences analysis of the detectable bat AAVs revealed a relatively large genetic diversity among the bat samples. This suggests that diverse AAVs may be widely distributed in bat populations as previous studies have reported^14,32^. However, further extensive work has to be performed to discover new AAV strains and their native bat species around the southeastern coastal regions of China.

The phylogenetic study of BtAAV-CXC1 with 26 other AAVs suggested that it was a novel AAV member of the genus Dependovirus. It was phylogenetically closer to mammalian AAVs compared to avian, waterfowl or snake AAVs. BtAAV-CXC1 shared a large branch with the previously reported Rp-BtAAV1, BtAAV1-YNM, and other known human or non-human primates AAVs. This included Adeno-associated virus A, Adeno-associated virus B, and all proven human AAVs (Fig. 3). Amino acid sequence analysis of the rep gene of this novel BtAAV-CXC1 showed 64.8% similarity with human AAV2. Similar results were obtained in a previous study^32^. In addition, several previous studies have indicated that mammalian and non-mammalian AAVs had a separate evolutionary path, except for a few avian, waterfowl and reptilian AAVs that were distantly related to mammalian AAVs. Bats are thought to be the original host for alpha coronaviruses and beta coronaviruses, while birds for gamma coronaviruses and delta coronaviruses^32,37,38^. The discovery of additional AAVs from bats and other mammals will contribute to a better understanding of the evolutionary origin and pathways of mammalian AAVs. Our study found that the co-occurrence of bat adenovirus and AAV in bat samples was as high as 33.7%, which was similar to the findings in Li *et.al*.’s study^14^. This may be because other viruses such as herpesvirus and vaccinia virus are essential for the replication of AAV^39^.

Adeno-associated viruses (AAVs) are frequently used viral vectors in biomedical research and human gene therapy. The clinical use of AAV vectors have been studied in detail. Over 160 phase I, II, and III clinical trials have been implemented since 1994, and have noticeably increased since 2011^40^. To date, substantial efforts have been dedicated on engineering strategies to develope novel AAV capsid variants with increased tropism for precise cell types with lower seroreactivity, and increased manufacturability. Our results revealed that the deduced Cap protein of BtAAV-CXC1 shared an average of 27.7% (ranging from 10.7 to 35.5%) pairwise amino acid identity with other mammalian AAVs. This suggests that the Cap protein of BtAAV-CXC1 may have a low cross-serological reactivity to known mammalian AAVs and hence could likely be considered to an substitutable vector for gene therapy.

In conclusion, our study of viruses in bat samples in southeast China identified novel AAVs and their distinct genetic diversity. This extends our knowledge of the diversity of bat AAVs and its evolution and emergence in humans and animals. However additional studies are warranted using larger samplings from different locations to further understand the global diversity of bat viruses.

## Materials and Methods

### Ethical approval

All procedures and protocols for sample collection and processing were approved by the Administrative Committee on Animal Welfare of the Institute of Zhejiang and Fujian CDC Veterinary and the Ethics Committee of the CDC of Easten Theater. All methods were performed in accordance with the relevant guidelines and regulations (Approval number: 2015009).

### Sample collection

Bat samples were collected from July 2015 to May 2018 from Zhejiang Province (including Dinghai and Daishan) and Fujian Province(including Changle, Lianjiang, Shishi and Xiamen), China. A total of 415 adult bats were captured alive at mountain caves with mist nets. Captured bats were carefully removed from the nets from each sampling location and were immediately euthanized and dissected. Bat details are shown in Table 1. Each sample (approximately 1 g of intestine tissue) was immediately transferred into viral transport medium and stored in liquid nitrogen prior to transportation to the laboratory^35^. Samples were stored at −80 °C until analyzed.

### RNA extraction and RT-PCR analysis

All samples were pooled and then underwent reverse transcription PCR analysis as previously described^36^. Briefly, each intestine sample (~0.1 g) was homogenized using a glass grinder with 10 volumes of SM buffer (50 mM Tris, 10 mM MgSO4, 0.1 M NaCl, pH7.5). The homogenates were centrifuged at 12,000 g for 10 min at 4 °C. The supernatants from each sample were passed through a 0.22 μm Pellicon II filter (Millipore, Billerica, MA). Viral RNA was extracted using the Viral RNA Mini Kit (Qiagen) following the manufacturer’s instructions. RNA was eluted in 30 μl RNase-free H_2_O and stored at −80 °C. Reverse transcription was performed using the 1st cDNA synthesis kit (TaKaRa), according to the manufacturer’s protocol. PCR was performed using cDNA with the following primers; forward primer 5′-AAGACCAACATCGCGGACATC-3′ and reverse primer 5′-TAGTTCTTGTTGWGRTGRTT-3′. The PCR reaction generated a 1000 nt fragment of the partial rep and cap gene. Primers were designed based on the nucleotide sequence of Rs-BtAAV1 (MF682927). The PCR amplification was performed in a 50 μl reaction mix containing 2 μl extracted cDNA, 10 μl PCR Buffer, 20 pmol of each primer, 4 μl dNTP (2.5 mM) and 1 μl Taq DNA polymerase (PrimeSTAR GXL, Takara). PCR conditions included 30 cycles of amplification, consisting of denaturation at 98 °C for 10 s, annealing at 65 °C for 15 s, extension at 68 °C for 1 min. Standard precautions were taken to avoid PCR contamination, and a negative control was included for each PCR assay. Amplified PCR products were sequenced in both directions using an ABI 3730 DNA Analyzer (Invitrogen, Beijing, China). Bat AAV identified from bat species *Rhinolophus pusillus* in the Fujiang province (designated BtAAV-CXC1) was selected for additional full-length genome sequencing (Supplementary Table S1).

### Sequencing of bat AAVs partial rep and cap genome regions

After PCR screening, all positive samples were PCR amplified that targeted 841 bp and 1100 bp fragments of rep and cap ORFs. Primer sequences are shown in Table 3. PCR amplification was performed in a 50 μl reaction mix containing 2 μl extracted cDNA, 10 μl PCR Buffer, 20 pmol of each primer, 4 μl dNTP(2.5 mM) and 1 μl *Taq* DNA polymerase (PrimeSTAR GXL, Takara). PCR conditions were; 30 cycles of amplification consisting of denaturation at 98 °C for 10 s, annealing at 65 °C for 15 s, extension at 68 °C for 1 or 2 min. Standard precautions were taken to avoid PCR contamination, and a negative control was included in each PCR assay. Amplified PCR products were sequenced in both directions using the ABI 3730 DNA Analyzer (Invitrogen, Beijing, China).

**Table 3:** Primers used for PCR amplification of the partial rep and cap sequences.

Primer	Sequences (5′–3′)	Amplification length
AAV-Rep1	GTCCCATTTGACGTGGAGGAACATC	841 bp
AAV-Rep2	GGGAGCCGCTTAGTCAGTTCAAAT	
AAV-Cap1	AAGACCAACATCGCGGAGGCCATC	1100 bp
AAS- Cap2	GCGTGGTTGTACTTGAGGTA	

### Sequence and phylogenetic analysis

Viral nucleotide and derived amino acid sequences were analyzed using the DNASTAR software package (Lasergene). Putative ORFs with a minimum size of 150 aa and a coding capacity were predicted using the NCBI ORF Finder (http://www.ncbi.nlm.nih.gov/gorf/gorf.html). Amplified sequences obtained by PCR amplification were aligned using BLAST in GenBank. Representative sequences of other known AAVs were downloaded from GenBank. MEGA7.0 software was used to construct the maximum-likelihood evolutionary tree of the nearly complete genome sequence of BtAAV-CXC1 or partial rep and cap sequences of bat AAVs. Bootstrap values were estimated using 1000 replicates on the ML substitution model^41^.

### Nucleotide sequence accession numbers

All sequences obtained in this study were deposited in GenBank under accession numbers MK391482 for BtAAV-CXC1 complete genome, MK357907, MK394009–MK394021 and MK394023–MK394056 for the 48 partial rep sequences of AAVs, MK394057–MK394080 for the 24 partial cap sequences of AAVs^42^.

## Supplementary information

Supplementary Table S1/S2/S3.
