# Supplementary material for: Distribution and genetic diversity of adeno-associated viruses in bats from coastal areas of Southeast China
Source: Sci Rep. 2020 Feb 28;10:3725. doi: 10.1038/s41598-020-60721-z (PMC7048818; doi:10.1038/s41598-020-60721-z)
Supplement: Supplementary file 1 — Supplementary Table S1/S2/S3. [file 41598_2020_60721_MOESM1_ESM.doc]

**Distribution and genetic diversity of adeno-associated viruses in bats from coastal areas of Southeast China**

Changqiang Zhu^1,#^, Chunhui Wang^1,#^, Jiahong Wu^3,#^ ,Fuqiang Ye^1,#^, Ruichen Lv^1^, Dan Hu^1^, Lele Ai^1^, Lu Yang^1^, Ting Wu^4^, Bo Li^1^, Chenxi Ding^1^, Bin Zhang^1^, Heng Lv^1^, Changjun Wang^2,*^, Weilong Tan^1,*^

**Supplementary Table S1** Primers used for PCR for amplification of the genome of BtAAV-CXC1

| **Primer** | **Sequences (5′-3′)** | **Amplification length** |
| --- | --- | --- |
| AAV-F1 | TTCGCTCGCTCGCTGGCTCGTT | 1100 bp |
| AAV-R1 | CTCCTGGTCCTCTTTGATCCATTCG |
| AAV -F2 | GTCCCATTTGACGTGGAGGAACATC | 1290bp |
| AAV -R2 | GGGAGCCGCTTAGTCAGTTCAAAT |
| AAV -F3 | AAGACCAACATCGCGGAGGCCATC | 1135 bp |
| AAV -R3 | GCGTGGTTGTACTTGAGGTA |
| AAV -F4 | TTGTTGATCACCCTCCAGATTGG | 1250 bp |
| AAV -R4 | GTGGAGGTGAGGTTGTTGGCGA |
| AAV -F5 | AAGATGGCTGMGGACGGCCAACAACTT | 1325 bp |
| AAV -R5 | GTTGTAGTTGTTGGTGTACTG |

**Supplementary Table S2** Rate of co-presence of bat adenoviruses and AAVs among 5 bat species.

| **Bat species** | **No. positive(adenoviruses )/no. tested(positive of adeno-associated virus )(%)** | | | | | | |
| --- | --- | --- | --- | --- | --- | --- | --- |
|  | **ZS** | **DS** | **XM** | **CL** | **SS** | **LJ** | **All locations** |
| *Rhinolophus pusillus* | 9/30(30.0) | 11/20(55.0) | 0/2(0.0) | 2/8(25.0) | 0/0 | 0/0 | 22/60(36.7) |
| *Rhinolophus ferrumequinum* | － | 1/5(20.0) | － | － | － | － | 1/5(20.0) |
| *Scotophilus kuhli* | － | － | 0/1(0) | － | － | － | 0/1(0) |
| *Myotis davidii* | 3/7(42.9) | － | － | 0/4(0) | － | － | 3/11(27.3) |
| *Myotis formosus* | － | 0/0 | － | － | － | － | 0/0 |
| All species | 12/37(28.7) | 12/25(17.2) | 0/3(11.1) | 2/12(21.8) | 0/0 | 0/0 | 26/77(33.7) |

*ZS: Dinghai; DS: Daishan; XM: Xiamen; CL: Changle; SS: Shishi; LJ:Lianjiang

**Supplementary Table S3** Prevalence of adenoviruses in the negative of adeno-associated virus among 5 bat species.

| **Bat species** | **No. positive**(adenoviruses )/no. tested(negative of adeno-associated virus )(%) | | | | | | |
| --- | --- | --- | --- | --- | --- | --- | --- |
|  | **ZS** | **DS** | **XM** | **CL** | **SS** | **LJ** | **All locations** |
| *Rhinolophus pusillus* | 11/72(15.3) | 7/71(9.9) | 0/17(0) | 2/36(5.6) | 3/35(5.6) | 0/24(0) | 23/255(9.0) |
| *Rhinolophus ferrumequinum* | － | 2/24(8.3) | － | － | － | － | 2/24(8.3) |
| *Scotophilus kuhli* | － | － | 0/7(0) | － | － | － | 0/7(0) |
| *Myotis davidii* | 1/20(5.0) | － | － | 1/7(14.3) | － | － | 2/27(7.4) |
| *Myotis formosus* | － | 4/25(16.0) | － | － | － | － | 4/25(16.0) |
| All species | 12/92(13.0) | 13/120(10.8) | 0/24(0) | 3/43(6.9) | 3/35(8.6) | 0/24(0) | 31/338(18.6) |

*ZS: Dinghai; DS: Daishan; XM: Xiamen; CL: Changle; SS: Shishi; LJ:Lianjiang
